# Observer uncertainties of soft tissue‐based patient positioning in IGRT

**DOI:** 10.1002/acm2.12817

**Published:** 2020-01-20

**Authors:** Taka‐aki Hirose, Hidetaka Arimura, Jun‐ichi Fukunaga, Saiji Ohga, Tadamasa Yoshitake, Yoshiyuki Shioyama

**Affiliations:** ^1^ Division of Radiology Department of Medical Technology Kyushu University Hospital Fukuoka Japan; ^2^ Faculty of Medical Sciences Kyushu University Fukuoka Japan; ^3^ Department of Clinical Oncology Graduate School of Medical Sciences Kyushu University Fukuoka Japan

**Keywords:** interobserver variation, intraobserver variation, prostate cancer image‐guided radiation therapy, PTV margin, soft‐tissue‐based patient positioning

## Abstract

**Purpose:**

There remain uncertainties due to inter‐ and intraobserver variability in soft‐tissue‐based patient positioning even with the use of image‐guided radiation therapy (IGRT). This study aimed to reveal observer uncertainties of soft‐tissue‐based patient positioning on cone‐beam computed tomography (CBCT) images for prostate cancer IGRT.

**Methods:**

Twenty‐six patients (7–8 fractions/patient, total number of 204 fractions) who underwent IGRT for prostate cancer were selected. Six radiation therapists retrospectively measured prostate cancer location errors (PCLEs) of soft‐tissue‐based patient positioning between planning CT (pCT) and pretreatment CBCT (pre‐CBCT) images after automatic bone‐based registration. Observer uncertainties were evaluated based on residual errors, which denoted the differences between soft‐tissue and reference positioning errors. Reference positioning errors were obtained as PCLEs of contour‐based patient positioning between pCT and pre‐CBCT images. Intraobserver variations were obtained from the difference between the first and second soft‐tissue‐based patient positioning repeated by the same observer for each fraction. Systematic and random errors of inter‐ and intraobserver variations were calculated in anterior–posterior (AP), superior–inferior (SI), and left–right (LR) directions. Finally, clinical target volume (CTV)‐to‐planning target volume (PTV) margins were obtained from systematic and random errors of inter‐ and intraobserver variations in AP, SI, and LR directions.

**Results:**

Interobserver variations in AP, SI, and LR directions were 0.9, 0.9, and 0.5 mm, respectively, for the systematic error, and 1.8, 2.2, and 1.1 mm, respectively, for random error. Intraobserver variations were <0.2 mm in all directions. CTV‐to‐PTV margins in AP, SI, and LR directions were 3.5, 3.8, and 2.1 mm, respectively.

**Conclusion:**

Intraobserver variability was sufficiently small and would be negligible. However, uncertainties due to interobserver variability for soft‐tissue‐based patient positioning using CBCT images should be considered in CTV‐to‐PTV margins.

## 1 INTRODUCTION

Prostate cancer is the most frequently diagnosed cancer in males. Approximately 1.3 million new cases of prostate cancer and 359 000 associated deaths were reported worldwide in 2018.^1^ Common approaches for treating localized prostate cancer include active surveillance, radical prostatectomy, radiotherapy, and hormonal therapy.^2^ Since external beam radiation therapy (a minimal dose of 72 Gy) showed similar biochemical relapse‐free survival rates of localized prostate cancer as radical prostatectomy,^3^ radiation therapy has an advantage for elderly people, who cannot undergo surgery due to complications.^2^, ^3^

In the current radiation therapy for prostate cancer, image‐guided radiation therapy (IGRT) with cone‐beam computed tomography (CBCT) images has been commonly used in clinical practice to increase the accuracy of patient positioning.^4^ Zelefsky et al. reported that intensity modulated radiotherapy (IMRT) with image‐guided patient positioning (IGPP) improved prostate‐specific antigen (PSA) outcomes and toxicities of organs at risk (OAR).^5^

Soft‐tissue‐based patient positioning with CBCT images^6^, ^7^, ^8^, ^9^ and intraprostatic fiducial marker‐based patient positioning^10^, ^11^, ^12^ have been used for more accurate target‐based‐patient positioning (TBPP). Soft‐tissue‐based patient positioning with CBCT images is a noninvasive approach and has the advantage of providing soft‐tissue information such as circumstances of targets and critical organs. However, there are uncertainties due to inter‐ and intraobserver variability,^6^, ^7^, ^8^, ^9^ which may influence clinical outcomes and OAR toxicities.

Several studies have assessed inter‐ and intraobserver variability in soft‐tissue‐based patient positioning.^6^, ^7^, ^8^, ^9^ Some studies have investigated the accuracy of soft‐tissue‐based patient positioning with CBCT images compared to patient positioning based on fiducial markers for patients undergoing external beam radiotherapy of the prostate.^6^, ^7^ Jereczek‐Fossa et al. used online CBCT positioning performed by a radiation oncologist immediately prior to treatment as reference for manual soft‐tissue‐based patient positioning reviewed by observers.^8^ To our knowledge, no observer studies have evaluated inter‐ and intraobserver variability in soft‐tissue‐based patient positioning using prostate contours on CBCT images.

We hypothesized that the uncertainties due to inter‐ and intraobserver variability for soft‐tissue‐based patient positioning using CBCT images would not be negligible. In this observer study, the uncertainties of soft‐tissue‐based patient positioning against contour‐based patient positioning were evaluated by systematic and random errors of inter‐ and intraobserver variability in anterior–posterior (AP), superior–inferior (SI), and left–right (LR) directions. Then, clinical target volume (CTV)‐to‐planning target volume (PTV) margins for the soft‐tissue‐based patient positioning were calculated from the systematic and random errors of the inter‐ and intraobserver variations.

## 2 MATERIALS AND METHODS

### 2.1 Overall evaluation scheme of uncertainties for soft‐tissue‐based patient positioning

**Figure 1:**
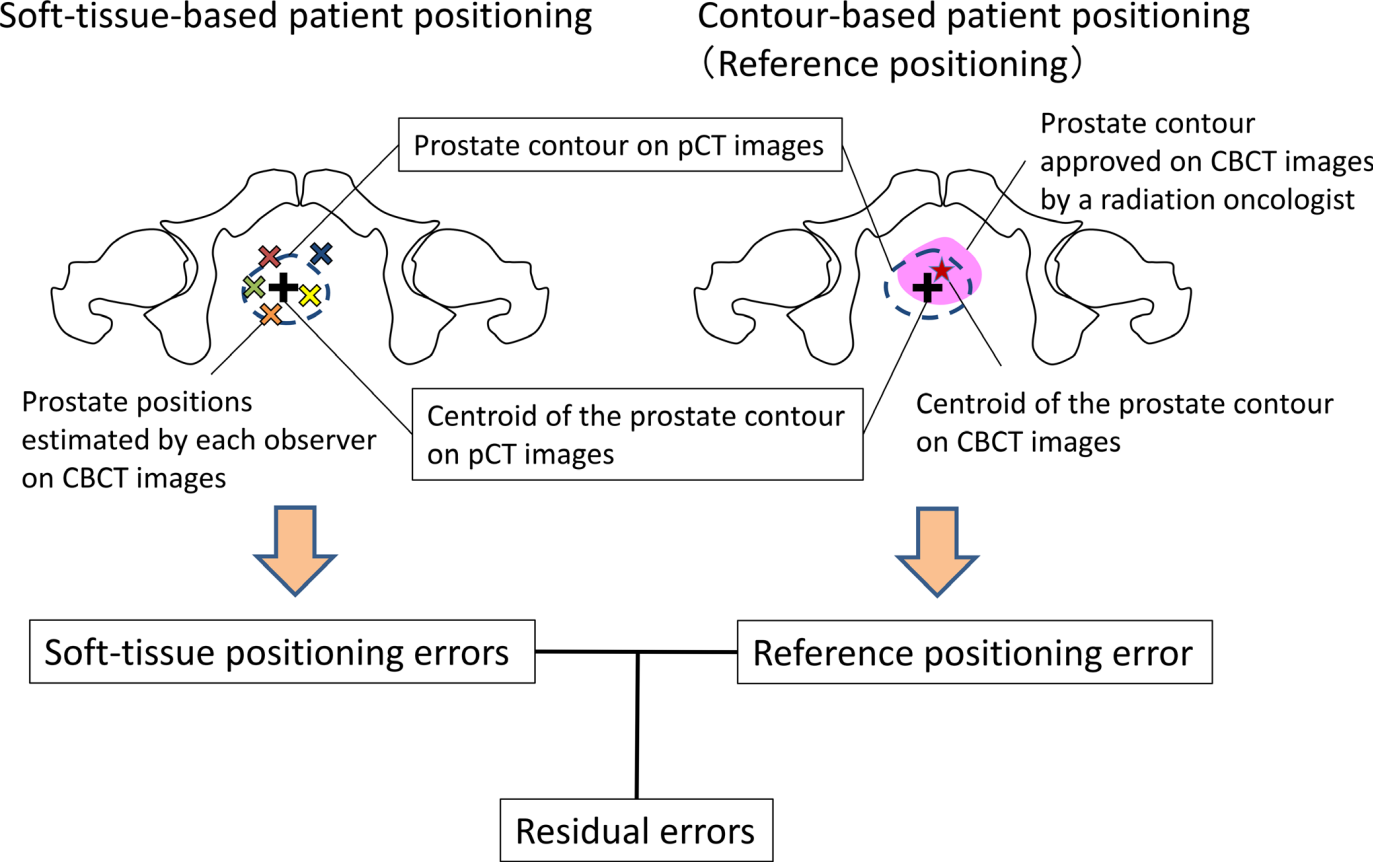
The evaluation scheme of residual errors by each observer on pre‐cone‐beam computed tomography image of a fraction.

The uncertainties for soft‐tissue‐based patient positioning were evaluated by inter‐ and intraobserver variations. The interobserver variations were obtained from residual errors, which denote differences between soft‐tissue positioning errors and reference positioning errors. The evaluation scheme of residual errors by each observer for a fraction is illustrated in Fig. 1. The reference positioning errors were obtained from prostate cancer location errors (PCLEs) of contour‐based patient positioning between planning CT (pCT) and pretreatment CBCT (pre‐CBCT) images. The PCLEs of contour‐based patient positioning indicate the centroid distance of prostate contours on pCT and pre‐CBCT images. The soft‐tissue positioning errors were measured from the PCLEs of soft‐tissue‐based patient positioning in the observer study, which were performed between pCT and pre‐CBCT images after an automatic bone‐based registration by six observers. Then, the intraobserver variations were evaluated from the differences between the first and second soft‐tissue‐based patient positioning repeated by the same observer for each fraction. Finally, CTV‐to‐PTV margins (hereafter PTV margins) were calculated from the systematic and random errors of the inter‐ and intraobserver variations in AP, SI, and LR directions.

### 2.2 Patient data and setup

This retrospective study was performed with the approval of the institutional review board of our hospital. Among patients treated between April 2012 and April 2013, 26 patients (median age: 68 yr; range: 56–83 yr; Stage: T1–T3a, N0, M0, median initial prostate‐specific antigen: 10.10 ng/mL; range: 3.56–99.17, median Gleason score (sum): 7; range: 6–9) who underwent IMRT without fiducial markers for prostate cancer (76 Gy in 38 fractions of 2 Gy) were selected. External immobilization with a simple knee support was used for pCT scans and treatments. Regarding bladder and rectum preparation, all patients were instructed to empty their bladders and rectums as much as possible and to drink 300 mL water 30 min before the scheduled treatment.

### 2.3 Image acquisition and equipment

The 26 patients were scanned using a pCT (Mx 8000, Philips Healthcare, Amsterdam, The Netherlands) with tube voltage of 120 kV, in‐plane pixel size of 0.98 mm, and slice thickness of 2.0 mm for treatment planning. Two hundred and four pre‐CBCT images were acquired (On‐Board‐Imager, Varian Medical Systems Inc., Palo Alto, USA) with half‐fan mode, with a tube voltage of 120 kVp, in‐plane pixel size of 1.17 mm, and slice thickness of 2.5 mm in 6–9 fractions (mean: 7) in 38 fraction treatment courses per patient, under a condition (‘‘pelvis’’ mode, 120 kV, 1040 mAs, and 365° acquisition angle range with a half‐fan bow tie filter). The CBCT images were acquired at the beginning of the week to reduce patient dose according to the principle of “as low as reasonably achievable (ALARA)”.

### 2.4 Measurement of soft‐tissue‐based patient positioning errors based on observer study

Soft‐tissue positioning errors were measured from PCLEs of soft‐tissue‐based patient positioning in the observer study performed between pCT and pre‐CBCT images after automatic bone‐based registration by six observers. The six observers with 3–14 yr of experience as radiation therapists (RTs) received prior training on the soft‐tissue‐based patient positioning procedures. Each observer first performed automatic registration to bone anatomy between pCT and pre‐CBCT images and then manually refined prostate position in AP, SI, and LR directions without rotation correction. The observers could utilize the transverse, sagittal, and coronal plane views and adjust window/level at any time. The contours of the prostate, seminal vesicles (SVs), rectum, and bladder delineated on pCT images were overlaid on pre‐CBCT images to aid soft‐tissue‐based patient positioning.^13^ The ARIA Oncology Information System (Varian Medical Systems Inc., Palo Alto, USA) off‐line review tool was used for matching pCT with pre‐CBCT images.

### 2.5 Determination of reference positioning errors based on contour‐based patient positioning

Contour‐based patient positioning based on the prostate contour delineated on pre‐CBCT images was used as a reference positioning for soft‐tissue‐based patient positioning. The reference positioning errors were obtained from the PCLEs of contour‐based patient positioning between the pCT and pre‐CBCT images, which indicate the centroid distance of the prostate contours on these images. Prostate contours on the pre‐CBCT images for each fraction were determined based on a consensus between the radiation oncologist (S.O.) and medical physicist (T.H.) using a commercially available radiation treatment planning (RTP) system (Eclipse version 10.0; Varian Medical Systems Inc., Palo Alto, USA).

### 2.6 Interobserver variations

Inter‐observer variations were evaluated from the residual errors, which denoted the differences between the soft‐tissue positioning errors and reference positioning errors for each fraction. The systematic error ($\varepsilon_{\mathrm{inter}}$) and random error ($\sigma_{\mathrm{inter}}$) for interobserver variations were calculated from the root mean square (RMS) of the residual errors by N observers, respectively, as

(1) $$\varepsilon_{\mathrm{inter}}=\sqrt{\frac{1}{N}\sum_{j=1}^{N}\varepsilon_{inter,\;j}^{2}}$$

and

(2) $$\sigma_{\mathrm{inter}}=\sqrt{\frac{1}{N}\sum_{j=1}^{N}\sigma_{inter,\;j}^{2}},$$

where N is the number of observers. $\varepsilon_{inter,j}$ and $\sigma_{inter,j}$ represent systematic and random errors of the residual errors for an observer *j*, respectively. The systematic error ($\varepsilon_{inter,j}$) and random error ($\sigma_{inter,j}$) for an observer *j* were given by

(3) $$\varepsilon_{inter,\;j}=\sqrt{\frac{1}{n}\sum_{i=1}^{n}{\left(m_{inter,\;i,\;j}-{\bar{m}}_{inter,\;j}\right)}^{2}}$$

and

(4) $$\sigma_{inter,\;j}=\sqrt{\frac{1}{n}\sum_{i=1}^{n}\sigma_{inter,\;i,\;j}^{2}},$$

respectively,where n is number of patients. $m_{inter,i,j}$ and ${\bar{m}}_{inter,j}$ represent the mean residual error of a patient i by an observer j and the mean residual error of all patients by the observer j, respectively. $\sigma_{inter,i,j}$ represents the SD of the residual error of a patient i by an observer j. $m_{inter,i,j}$ and ${\bar{m}}_{inter,j}$ are given by

(5) $$m_{inter,\;i,\;j}=\frac{1}{F}\sum_{k=1}^{F}d_{inter,\;i,\;j,\;k},\;{\bar{m}}_{inter,\;j}=\frac{1}{n}\sum_{i=1}^{n}m_{inter,\;i,\;j},$$

where F is the number of fractions. $d_{inter,i,j,k}$ represents the residual error at a fraction k of a patient i by an observer j, which denotes the difference between a soft‐tissue positioning error and reference positioning error at each fraction.

### 2.7 Intraobserver variations

To further explore the effect of intraobserver variations in soft‐tissue‐based patient positioning on prostate IGRT, each observer repeated the soft‐tissue‐based patient positioning process for the same cases 3 months later. An intraobserver variation was evaluated as the difference between the first and second soft‐tissue‐based patient positionings repeated by an observer at each fraction of a patient. Five observers participated in the observer study with ten patients. The systematic error ($\varepsilon_{\mathrm{intra}}$) and random error ($\sigma_{\mathrm{intra}}$) for intraobserver variations were calculated from the RMS of the intraobserver errors by N observers, respectively, as

(6) $$\varepsilon_{\text{int}ra}=\sqrt{\frac{1}{N}\sum_{j=1}^{N}\varepsilon_{intra,\;j}^{2}}$$

and

(7) $$\sigma_{\text{int}ra}=\sqrt{\frac{1}{N}\sum_{j=1}^{N}\sigma_{intra,\;j}^{2}},$$

where N is the number of observers. $\varepsilon_{intra,j}$ and $\sigma_{intra,j}$ represent systematic and random errors of the intraobserver errors for an observer *j*, respectively. The systematic error ($\varepsilon_{intra,j}$) and random error ($\sigma_{intra,j}$) for an observer *j* were given by

(8) $$\varepsilon_{intra,\;j}=\sqrt{\frac{1}{n}\sum_{i=1}^{n}{\left(m_{intra,\;i,\;j}-{\bar{m}}_{intra,\;j}\right)}^{2}}$$

and

(9) $$\sigma_{intra,\;j}=\sqrt{\frac{1}{n}\sum_{i=1}^{n}\sigma_{intra,\;i,\;j}^{2}},$$

where n is number of patients. $m_{intra,i,j}$ and ${\bar{m}}_{intra,j}$ represent the mean intraobserver error of a patient i by an observer j and mean intraobserver error of all patients by the observer j, respectively. $\sigma_{intra,i,j}$ represents SD of the intraobserver errors of a patient i by the observer j. $m_{intra,i,j}$ and ${\bar{m}}_{intra,j}$ are given by

(10) $$m_{intra,\;i,\;j}=\frac{1}{F}\sum_{k=1}^{F}d_{intra,\;i,\;j,\;k},\;{\bar{m}}_{intra,\;j}=\frac{1}{n}\sum_{i=1}^{n}m_{intra,\;i,\;j},$$

where F is the number of fractions. $d_{intra,i,j,k}$ represents the intraobserver error at a fraction k of a patient i by an observer j, which denotes the difference between the first and second soft‐tissue‐based patient positioning repeated by an observer at each fraction of a patient.

### 2.8 PTV margin calculations

Planning target volume margins were calculated from the systematic and random errors of interobserver and/or intraobserver variations using the van Herk’s margin formula,^14^ as follows:

(11) $$PTV\;margin=2.5\sqrt{\varepsilon_{\mathrm{inter}}^{2}+\varepsilon_{\mathrm{intra}}^{2}}+0.7\sqrt{\sigma_{\mathrm{inter}}^{2}+\sigma_{\mathrm{intra}}^{2}}$$

This formula was derived based on a dose‐population histogram to deliver at least 95% of a prescribed dose to 90% of a patient population.

## 3 RESULTS

### 3.1 Residual errors

**Figure 2:**
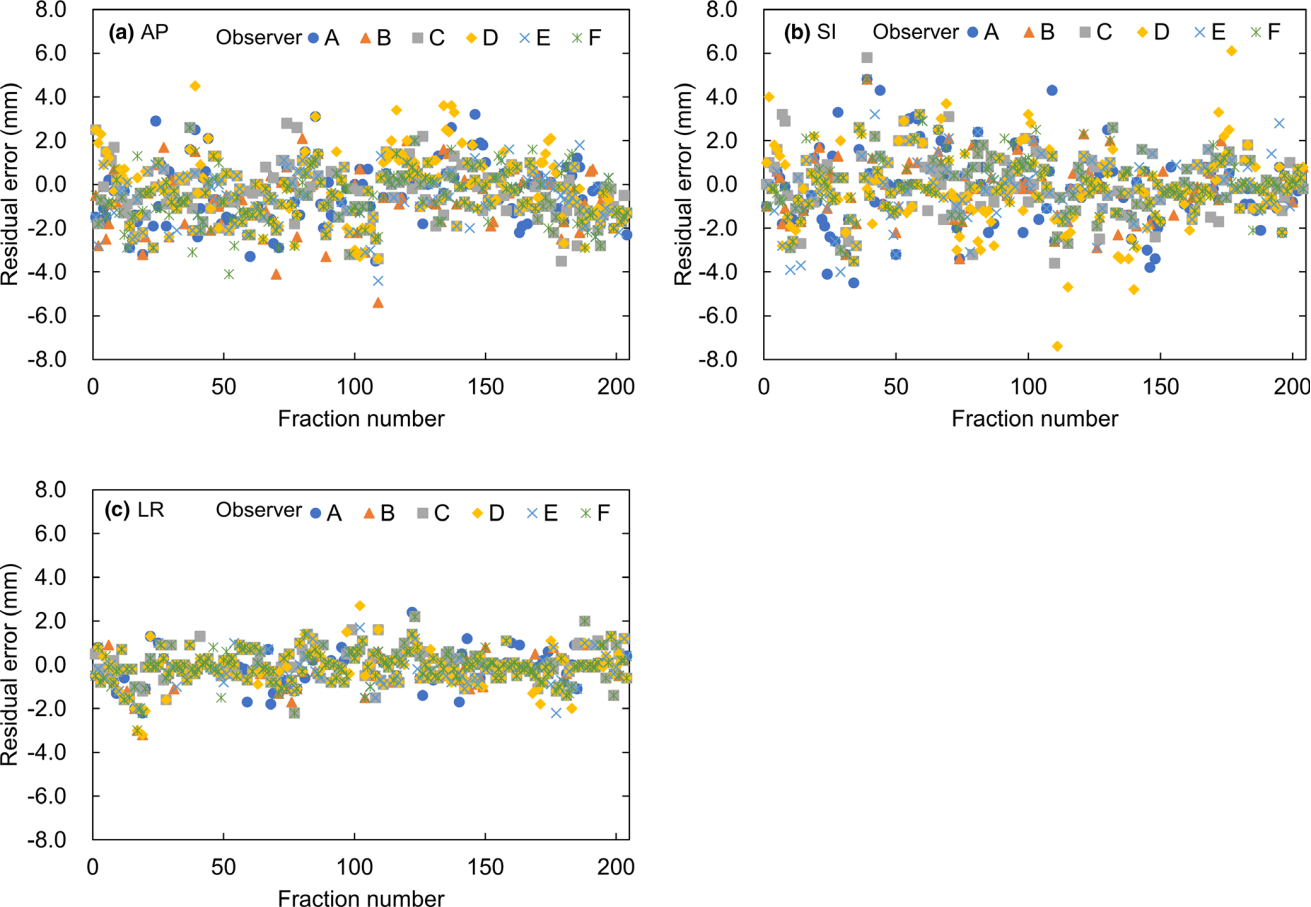
Residual errors between soft‐tissue positioning errors and reference positioning errors by six observers in anterior–posterior, superior–inferior, and left–right directions.

Figure 2 shows the residual errors between soft‐tissue positioning errors and reference positioning errors for 204 fractions for all patients by six observers in AP, SI, and LR directions. Variations of the residual error were largest in SI direction and smallest in LR direction.

### 3.2 Interobserver variations

**Figure 3:**
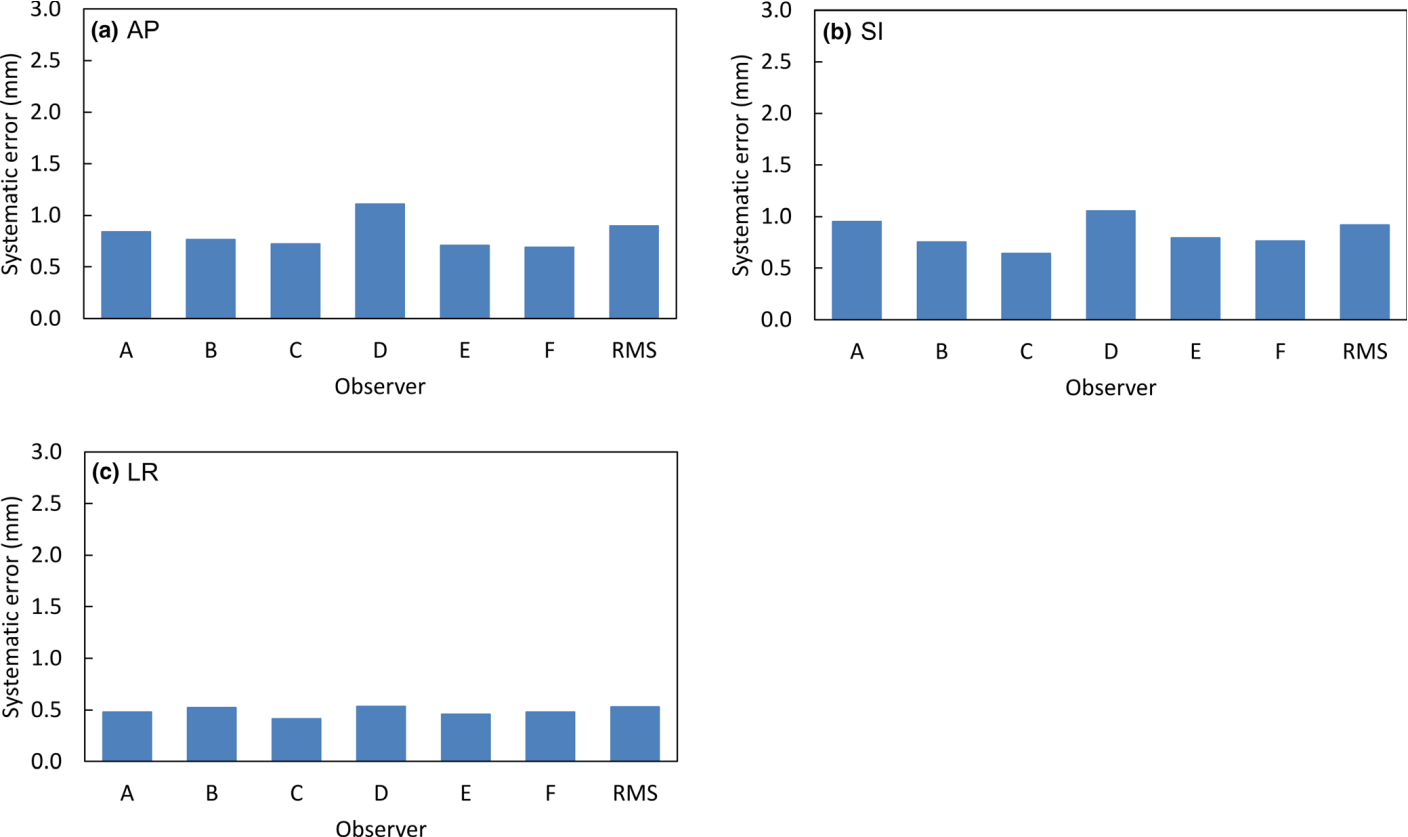
Systematic errors of interobserver variations by six observers in anterior–posterior, superior–inferior, and left–right directions.

Figure 3 shows the systematic errors of interobserver variations calculated from the residual errors for each observer in AP, SI, and LR directions. The systematic errors of interobserver variations for each observer in AP, SI, and LR directions were 0.7–1.1 mm, 0.6–1.1 mm, and 0.4–0.5 mm, respectively. The RMSs of the systematic errors for interobserver variation by six observers were 0.9, 0.9, and 0.5 mm in AP, SI, and LR directions.

**Figure 4:**
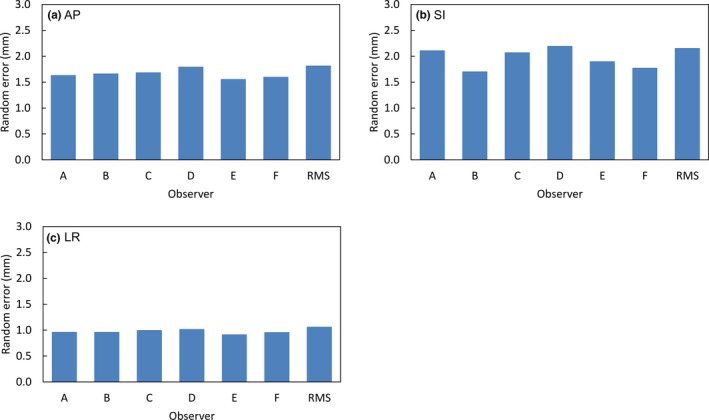
Random errors of interobserver variations by six observers in anterior–posterior, superior–inferior, and left–right directions.

Figure 4 shows the random errors of interobserver variations for each observer in AP, SI, and LR directions. The random errors of the interobserver variations for each observer in AP, SI, and LR directions were 1.6–1.8 mm, 1.7–2.2 mm, and 0.9–1.0 mm, respectively. The RMSs of the random errors for interobserver variations by the six observers were 1.8, 2.2, and 1.1 mm in AP, SI, and LR directions.

### 3.3 Intraobserver variations

The systematic and random errors of intraobserver variations calculated as the difference between the first and second soft‐tissue‐based patient positioning repeated by each observer for each fraction were <0.2 mm in AP, SI, and LR directions.

### 3.4 PTV margins

**Figure 5:**
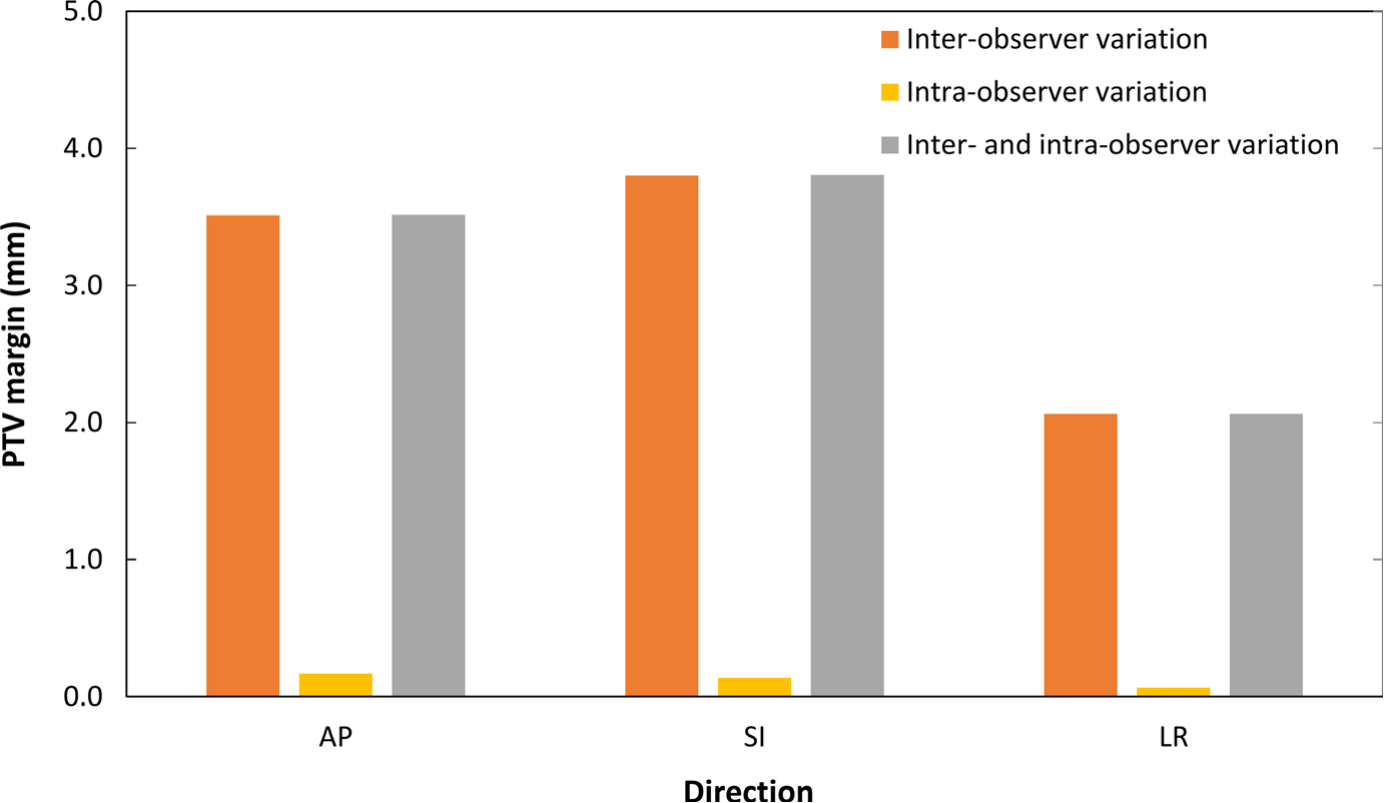
Planning target volume margins calculated from the systematic and random errors of interobserver and/or intraobserver variations in anterior–posterior, superior–inferior, and left–right directions.

Figure 5 shows the PTV margins calculated from the systematic and random errors of interobserver and/or intraobserver variations in AP, SI, and LR directions. The PTV margins required for interobserver variations in AP, SI, and LR directions were 3.5, 3.8, and 2.1 mm, respectively, while the PTV margins required for the intraobserver variations in all directions were <0.3 mm. The PTV margins considering both interobserver and intraobserver variations in AP, SI, and LR directions were 3.5, 3.8, and 2.1 mm, respectively. The impact was too small to consider the intraobserver variations on PTV margins.

## 4 DISCUSSION

This study evaluated the uncertainties for soft‐tissue‐based patient positioning by multiple observers against contour‐based patient positioning on pre‐CBCT images in IGRT for prostate cancer. The interobserver variations in AP, SI, and LR directions were 0.9, 0.9, and 0.5 mm for systematic error, and 1.8, 2.2, and 1.1 mm for random error, respectively. The systematic and random errors of the intraobserver variations were <0.2 mm. Therefore, interobserver variations were the largest source of uncertainties for soft‐tissue‐based patient positioning. Furthermore, the PTV margins considering both interobserver and intraobserver variations in AP, SI, and LR directions were 3.5, 3.8, and 2.1 mm, respectively.

Regarding interobserver variation, Moseley et al. defined fiducial marker positions using megavoltage (MV) imaging as the truth for soft‐tissue‐based patient positioning to evaluate systematic and random errors for interobserver variations by soft‐tissue‐based patient positioning on CBCT images.^6^ The interobserver variations in AP, SI, and LR directions were 1.61, 2.21, and 0.61 mm for systematic error, and 2.86, 2.85, and 1.50 mm for random error, respectively. In our results, as shown in Figs. 3 and 4, the interobserver variations for soft‐tissue‐based patient positioning in AP, SI, and LR directions were 0.9, 0.9, and 0.5 mm for systematic error, and 1.8, 2.2, and 1.1 mm for random error, respectively. There is no ground truth for prostate position such as a fiducial marker in soft‐tissue‐based patient positioning. Therefore, contour‐based patient positioning using prostate contour, based on a consensus between the radiation oncologist and medical physicist on pre‐CBCT images, was used as reference positioning for soft‐tissue‐based patient positioning in this study. Despite the differences in these reference positionings, interobserver variations were smaller than those in previous study. Thus, our results indicated interobserver consistency and good agreement between soft‐tissue‐based patient positioning and contour‐based patient positioning. We consider that sufficient observer training led to consistency and consensus regarding patient positioning by reducing interobserver variations. Furthermore, calcifications as anatomical landmarks in the prostate of some patients might have contributed to the accuracy of patient positioning.^15^

Morrow et al. reported that intraobserver variations were within 1 mm in manual soft‐tissue‐based patient positioning with kV‐CBCT images for prostate radiotherapy.^9^ In this study, the systematic and random errors of intraobserver variations were <0.2 mm. The PTV margins required for intraobserver variations were within 0.3 mm, and the impact of the uncertainties due to intraobserver variations on PTV margin was sufficiently small. Therefore, the influence of intraobserver variations on soft‐tissue‐based patient positioning would be negligible.

The clinical applicability of PTV margins was investigated in a simulation of IMRT plans. The IMRT plans for prostate cancer using the proposed PTV margins of 3.5, 3.8, and 2.1 mm in AP, SI, and LR directions, were created for 30 validation patients. The treatment plans were then compared with the original plans used in clinical practice, in which a PTV margin of 6 mm expansion in all directions, except 4 mm posteriorly, was added. Table 1 indicates target and OAR dose parameters for 30 validation patients in the treatment plans with PTV_CL_ used in clinical practice and PTV_ST_ obtained from the PTV margins required for soft‐tissue‐based patient positioning in this study. The conformity index (CI) and homogeneity index (HI) were used for dose evaluation of the plans with PTV_CL_ and PTV_ST_. These dose evaluation indices were defined based on ICRU report 83^16^ as follows:

(12) $$CI=\frac{V_{\mathrm{PTV}}}{V_{PD98\%}}$$

and

(13) $$HI=\frac{D2-D98}{D50},$$

where V_PTV_ is the volume of PTV, and V_PD98%_ is the volume receiving 98% of the prescribed dose. D2, D98, and D50 mean the minimum dose that covers 2%, 98%, and 50% of the PTV, respectively. Dose conformity of PTV was significantly greater in the treatment plan with PTV_ST_ than that with PTV_CL_. There were no statistically significant differences in CTV coverage indices of D98 and D95 between the treatment plans with PTV_ST_ and PTV_CL_. The OAR doses in the treatment plans with PTV_ST_ showed lower value than those with PTV_CL_ in all dose parameters. The dose coverages to CTV and PTV larger than 90% in the treatment plan with proposed PTV_ST_ were acceptable in clinical practice, and the small PTV margins contributed to OAR dose reduction. Therefore, the PTV margin estimated in this study would be applicable in clinical practice. However, attention should be paid to the determination of PTV margins, because small PTV margins of less than 3 mm may cause lower CTV coverages due to organ deformations and volume changes in actual treatment.^17^

**Table 1:** Comparison of target and OAR dose parameters for 30 validation patients in the treatment plans with PTV_CL_ used in clinical practice and PTV_ST_ obtained from the PTV margins required for soft‐tissue‐based registration in this study.

Volume of interest	Parameter	PTV_CL_	PTV_ST_	*P* value
		Mean ± SD	Mean ± SD	
PTV_CL_ or PTV_ST_	D98	91.75 ± 1.31	91.71 ± 1.31	n.s.*
	D95	95.93 ± 0.74	96.06 ± 0.88	n.s.*
	CI	1.02 ± 0.03	0.99 ± 0.02	<0.05
	HI	0.11 ± 0.01	0.11 ± 0.01	n.s.*
CTV	D98	99.18 ± 0.29	99.19 ± 0.23	n.s.*
	D95	99.62 ± 0.22	99.60 ± 0.18	n.s.*
Rectum	V40	20.33 ± 4.69	14.85 ± 4.15	<0.05
	V60	9.20 ± 2.80	5.50 ± 1.99	<0.05
	V65	6.82 ± 2.24	3.77 ± 1.50	<0.05
	V70	4.09 ± 1.55	2.01 ± 0.96	<0.05
	V75	0.63 ± 0.73	0.20 ± 0.29	<0.05
Bladder	V40	25.12 ± 8.85	19.33 ± 8.37	<0.05
	V70	10.40 ± 3.93	6.68 ± 3.34	<0.05
	V75	7.72 ± 3.01	4.47 ± 2.33	<0.05

CI, conformity index; CTV, clinical target volume; Dn, dose received by n% of the volume; HI, homogeneity index; OAR, organs at risk; PTV, planning target volume; Vm, volume receiving at least m Gy dose.

* n.s. = not significant (*P* > 0.05).

Previous studies often used intraprostatic fiducial markers as reference for soft‐tissue‐based patient positioning with CBCT images in IGRT for prostate cancer.^6^, ^7^ However, in‐migration of these fiducial markers has been observed with a reduction in prostate volume during radiation treatment courses.^18^, ^19^, ^20^ In addition, Loh et al. reported a high rate of symptomatic infection with fiducial marker implantation and concluded that noninvasive approaches for prostate IGRT, such as CBCT, should be considered.^21^ The present study has the advantage that the uncertainties of soft‐tissue‐based patient positioning were evaluated on pre‐CBCT images without fiducial markers.

If same therapists perform soft‐tissue‐based patient positioning during the entire course of the treatment, the intraobserver variability would be <0.2 mm, which may be negligible. However, there could remain an uncertainty specific to each observer. Therefore, the uncertainty of the observer should be taken into account for calculating PTV margins. Nevertheless, the observer‐specific uncertainties can also be evaluated by comparing with the contour‐based patient positioning proposed in this study.

Recently, a commercial on‐board CBCT equipped with an iterative image reconstruction function has been available. The iterative image reconstruction algorithm can improve the CBCT image quality by reducing noise and artifacts.^22^ Morrow et al. recommended the use of modalities with higher image quality for IGRT, because the image quality affects the reproducibility of the manual image registration and the daily patient positioning shifts for IGRT.^9^ Therefore, if higher image quality CBCT images were employed during the treatment course, it would affect observer uncertainties of the soft‐tissue‐based patient positioning and contribute to reducing PTV margins.

This study had several limitations. First, it was evaluated only under limited conditions. There were only a few cases and observers considered for the evaluation of observer uncertainties for soft‐tissue‐based patient positioning in this study. More cases and observers for analyzing the uncertainties for soft‐tissue‐based patient positioning could yield more adequate PTV margins. An evaluation using more cases and all fractions is required in future work.

Furthermore, SVs were not evaluated even in intermediate‐ and high‐risk cases. Liang et al. explored interfractional prostate and SV motion,^23^ reporting that the SVs could move independently from the prostate with a motion magnitude larger than that of the prostate. These organ motions and deformations may have influenced the results of the observer study when SV positions were considered in soft‐tissue‐based patient positioning. Additional assessment of soft‐tissue‐based patient positioning including SVs in intermediate‐ and high‐risk cases is required.

Another limitation of this study was the uncertainty in prostate contours delineated manually on pCT and pre‐CBCT images, which may include delineation errors. The impact may be larger for low contrast images such as CBCT images. Hence, to reduce delineation errors, at least intra‐ and interobserver delineation variability, all prostate contours on pre‐CBCT images were determined based on consensus between the same radiation oncologist and medical physicist. However, Gardner et al. reported similar variability in human observer contours between pCT and CBCT images.^24^ Therefore, we used prostate contours delineated manually on pre‐CBCT images as a reference based on the assumption of a sufficiently small impact of CBCT image quality on delineation errors.

## 5 CONCLUSIONS

In conclusion, the results of the present study revealed the uncertainties for soft‐tissue‐based patient positioning by inter‐ and intraobserver variability on pre‐CBCT images in IGRT for prostate cancer. Interobserver variations in AP, SI, and LR directions were 0.9, 0.9, and 0.5 mm, respectively, for systematic error and 1.8, 2.2, and 1.1 mm, respectively, for random error. The systematic and random errors of intraobserver variations were <0.2 mm in all directions. The PTV margins considering both interobserver and intraobserver variations in AP, SI, and LR directions were 3.5, 3.8, and 2.1 mm, respectively. Consequently, while intraobserver variability was sufficiently small and would be negligible, uncertainties due to interobserver variability for soft‐tissue‐based patient positioning using CBCT images should be considered in CTV‐to‐PTV margins.

## CONFLICT OF INTEREST

No conflict of interest.
